# Relationship between Body Mass Index, Cardiorespiratory and Musculoskeletal Fitness among South African Adolescent Girls

**DOI:** 10.3390/ijerph15061087

**Published:** 2018-05-28

**Authors:** Emmanuel Bonney, Gillian Ferguson, Bouwien Smits-Engelsman

**Affiliations:** 1 Department of Health and Rehabilitation Sciences, University of Cape Town, Cape Town 7700, South Africa; gillian.ferguson@uct.ac.za (G.F.); bouwiensmits@hotmail.com (B.S.-E.); 2 Department of Physiotherapy, School of Biomedical & Allied Health Sciences, University of Ghana, Accra, Ghana

**Keywords:** cardiorespiratory fitness, musculoskeletal fitness, body mass index, adolescents, low-income settings

## Abstract

*Background*: Cardiorespiratory and musculoskeletal fitness are important health indicators that support optimal physical functioning. Understanding the relationship between body mass index and these health markers may contribute to the development of evidence-based interventions to address obesity-related complications. The relationship between body mass index, cardiorespiratory and musculoskeletal fitness has not been well explored, particularly in female adolescents. The aim of this study was to investigate the association between body mass index, cardiorespiratory and musculoskeletal fitness among South African adolescent girls in low-income communities. *Methods*: This cross-sectional study included 151 adolescent girls, aged 13–16 years. Cardiorespiratory fitness was measured using the 20 m shuttle run test and musculoskeletal fitness was assessed using a variety of field-based tests. Height and weight were measured with standardised procedures and body mass index (BMI) was derived by the formula [BMI = weight (kg)/height (m)^2^]. Participants were categorised into three BMI groups using the International Obesity Task Force age- and gender-specific cut-off points. Pearson correlations were used to determine the association between body mass index, cardiorespiratory fitness and measures of musculoskeletal fitness at *p* ≤ 0.05. *Results*: Overweight and obese girls were found to have lower cardiorespiratory fitness, decreased lower extremity muscular strength, greater grip strength, and more hypermobile joints compared to normal-weight peers. BMI was negatively associated with cardiorespiratory fitness and lower extremity muscular strength. *Conclusions*: The findings indicate that increased body mass correlates with decreased cardiorespiratory and musculoskeletal fitness. Interventions should be developed to target these important components of physical fitness in this demographic group.

## 1. Introduction

Childhood obesity is a global health priority [1,2]. Growing evidence indicates that childhood obesity rates seem to be leveling-off in high-income societies. However, estimates in low- and middle-income countries (LMIC) have increased rapidly over the past few years [3,4]. Research shows that about 8–13% of boys and girls in low-income communities are overweight or obese [3]. The prevalence of overweight and obesity combined among South African children is slightly above 15% [5]. Within the South African context, girls have higher prevalence of overweight and obesity than boys [5,6,7,8,9]. These statistics are worrying as they pose serious threats to the country’s health care system [4]. Obesity is associated with health complications including hypertension, type 2 diabetes and orthopaedic problems [10,11,12].

Further, obesity is known to impair physical fitness and academic performance [13,14,15]. Several factors have been postulated to explain how obesity promotes decreased physical fitness. First, obese children have low levels of physical activity compared to non-obese peers [16]. Consequently, they have less opportunity to develop motor skills which causes further participation restrictions and muscle deconditioning [17,18,19]. Compared to non-obese peers, obese children tend to avoid weight-bearing tasks (e.g., walking, running) due to the high energy cost associated with such activities. This leads to poor musculoskeletal and cardiorespiratory fitness [20]. Secondly, it has been suggested that obesity-related fitness impairments are caused by neuromuscular dysfunction resulting from metabolic imbalance [21]. The association between obesity and academic performance is purported to be influenced by factors such as poor peer-relationships, low-self-esteem, and reduced cognitive abilities [13]. Though obesity is known to negatively impact physical performance, the nature of the relationship between body mass index (BMI) and aspects of physical fitness is less clear, particularly among female adolescents from low socioeconomic backgrounds. Given the current interest in adolescent health [22] and the increased prevalence of adolescent obesity in LMIC [3], examining the association between BMI, cardiorespiratory and musculoskeletal fitness may provide opportunities for identifying effective interventions for reducing obesity-related complications. 

Several studies have documented an inverse relationship between BMI and cardiorespiratory fitness [10,14,23,24,25,26]. Higher BMI has a negative influence on musculoskeletal fitness [27]. Improved cardiorespiratory and musculoskeletal fitness (MSF) are each associated with better health outcomes [13,14,15]. MSF encompasses those components of physical fitness responsible for successful execution of motor tasks such as walking and running [28], and includes measures such as muscular strength and endurance, flexibility and joint mobility [29]. Lower levels of MSF are associated with higher BMI in school-aged children [6]. Though these trends are thought to be gender-dependent [30], the majority of existing data are based on preadolescent children of both sexes. Evidence of the association between BMI, cardiorespiratory and MSF in female adolescents is limited.

Previous studies were conducted among children from high-income countries. It is unclear if findings from such studies can be extrapolated to populations in low-income contexts. It is possible that the association between these factors might differ for populations in different settings. Without better understanding of how BMI relates to cardiorespiratory and MSF, health professionals may be less effective at promoting physical health in adolescent populations. Examining the relationship between BMI, cardiorespiratory and MSF in female adolescents would extend the body of knowledge. Therefore, the purpose of this study was to determine the association between BMI, cardiorespiratory and MSF among female adolescents attending school in a low-income community of Cape Town, South Africa. It was hypothesised that participants with higher BMI would have decreased cardiorespiratory and MSF than normal-weight peers. 

## 2. Materials and Methods

### 2.1. Study Design and Setting

This cross-sectional study analysed baseline data of a larger school-based intervention study designed to improve motor competence and physical fitness among South African adolescent girls. Female adolescents attending a quintile three high school in a low-income area of Cape Town, South Africa, comprised the sample. In South Africa, schools are classified into five categories called quintiles (Q1–Q5), based on their poverty score or quintile rank [9]. The quintile rank of a school is determined on the basis of the poverty level of the community in which it is located [9]. Schools in lower quintiles (Q1–Q3) are the poorest compared to higher quintile schools (Q4 and Q5). Because of this, children in lower quintile schools do not pay school fees as they receive financial support from the government. The school in which the present study was conducted was purposively selected as it is located in the largest township community in Cape Town and has relatively large number of students. Secondly, it is a beneficiary of the University of Cape Town’s (UCT) Schools Improvement Initiative (SII). The SII is one of the numerous social responsive programmes that draws on the university community’s resources to improve learning experiences of school-aged children living in low-income settings. Parents provided written informed consent and each girl gave child assent before participation. Ethical approval for this study was granted by the Human Research and Ethics Committee of the University of Cape Town (ref# 232/2016). Permission was sought from the Western Cape Education Directorate and senior management of the school.

### 2.2. Participants

One hundred and fifty-one (151) female students (aged 13–16 years) participated in this study. Participants were recruited using convenience sampling techniques. Participation was entirely voluntary, and participants were free to withdraw at any time. All the female students in grades 8–10 were invited to participate in the study. Participants with any physical disability (e.g., fractures) that hindered their participation in testing procedures were not included. In addition, girls whose parents reported cardiovascular and neuromuscular complications were excluded. Participants were isiXhosa speaking (isiXhosa is one of the native languages of South Africa) South African adolescent girls. The fact that participants attended a quintile three school confirmed they had lower socioeconomic backgrounds. Assessments were carried out within the school environment and occurred during regular physical education hours. All assessments were performed by trained research assistants (physiotherapists and physiotherapy students) with experience working with children and adolescents.

### 2.3. Measures

#### 2.3.1. Anthropometric Measures

Height and weight were measured, and BMI was calculated using the formula [BMI = weight (kg)/height (m)^2^]. Height was measured to the nearest 0.1 cm with a wall mounted tape measure while weight was measured to the nearest 0.1 kg using an electronic scale. BMI scores were categorised into normal-weight, overweight and obese groups using BMI definitions proposed by the International Obesity Task Force [31]. Demographic data including age and grade were collected by self-report.

#### 2.3.2. Cardiorespiratory Fitness

The 20 m shuttle run test was used to measure cardiorespiratory fitness. The 20 m shuttle run test was performed in accordance with standardised procedures [32]. Participants ran from one cone to another 20 m apart, while keeping pace with a pre-recorded beep [33]. The beep increased after every minute and participants were told to keep up with the beep for as long as possible [33]. When a participant failed to reach the appropriate cone within the stipulated time on two consecutive occasions or could no longer maintain the pace, the test was stopped. The number of shuttles (level) was recorded and used in the analysis.

#### 2.3.3. Muscular Strength

##### Upper and Lower Extremity Isometric Strength

This was assessed with a handheld dynamometer (the MicroFET2, Hogan Health Industries Inc., Salt Lake City, UT, USA) using the break test [34]. The elbow flexors and extensors, knee extensors, ankle plantarflexors and dorsiflexors were assessed. Participants were tested in supine (for the muscles of the elbow and ankle) and in seated position (for the knee extensors). Testing was conducted in accordance with published protocol [34]. The handheld dynamometer is viewed as a valid and reliable measure of isometric muscle strength [35,36]. The Intraclass Correlation Coefficient (ICC) for the MicroFet2 test-retest reliability ranges from 0.73 to 0.91 [37].

##### Handgrip Strength

Grip strength was measured using the Jamar hydraulic hand dynamometer [38]. The test was performed in accordance with standard procedures [39]. Three trials were conducted for each muscle group and the best score was used in the analysis.

#### 2.3.4. Flexibility

The sit-and-reach (SR) test was used to assess flexibility [40]. Participants were tested individually under supervision. Each participant was required to sit with their feet approximately hip-wide apart against a wooden testing box, with their knees in extension. Next, they were asked to place their right hand over the left, and slowly reach forward as far as possible by sliding their hands along the measuring rule. The farthest distance reached was recorded to represent the participant’s flexibility. Three trials were conducted, and the best score was reported. The SR is reported to have moderate criterion validity (r = 0.32–0.67) in young people [41]

#### 2.3.5. Joint Mobility

The Beighton test was used to assess joint hypermobility [42]. The test consists of four passive range of motion (ROM) items (assessed bilaterally) and one active forward flexion task. Briefly, the test involved dorsiflexion of the little finger beyond 90°, hyperextension of the elbow beyond 90°, hyperextension of the knee, apposition of the thumb to the flexor side of the forearm and forward flexion of the trunk with the knees straight, so that the palms of the hand can touch the floor easily. Participants were given a numerical score of 0 to 9, one point being awarded for a positive test. For this test, higher scores represent hypermobile joints. Although the Beighton test has not been formally evaluated for its psychometric properties in this population, it is considered as a valid test of joint mobility in children [42]. Additionally, joint ROM was assessed to the nearest 1-degree with a standard 2-legged 360-degree type Collehon extendable goniometer (01135; Lafayette Instrument Company, Lafayette, IN, USA).

#### 2.3.6. Musculoskeletal Complaints

Musculoskeletal complaints were assessed using a self-administered questionnaire designed for this study. The questionnaire included two closed ended questions that sought to determine the presence of pain in specific anatomical structures (Questions 1. “Do you have pains in your joints?” 2. “Do you have pains in your muscles?”). Participants responded to each of the above questions with either a yes or no. A third question sought to establish the severity of pain (3. How severe is your pain today?). For this question, participants were required to rate their pain intensity (severity) on a 10-point (0 = no pain, 10 = most severe pain) smiley faces sheet adapted from the Faces Pain Scale (FPS-R) [43].

### 2.4. Statistical Analysis

Data analyses were performed using SPSS (version 24.0, SPSS Inc., Chicago, IL, USA). Descriptive statistics including mean, standard deviation and percentages were used to summarise descriptive data. Differences in age and anthropometric characteristics were determined using the one-way Analysis of variance (ANOVA). For comparison, participants were initially categorized into one of three groups (normal-weight, overweight and obese) as defined by the previously mentioned guidelines [31]. Further, a one-way analysis of variance analysis was performed to compare the differences in cardiorespiratory fitness, muscular strength, flexibility and joint mobility among the three groups. Bonferroni post hoc pairwise comparison was used to extrapolate statistically significant differences. In addition, *χ*^2^ analysis was completed to compare the differences in musculoskeletal complaints and three sub-items of the Beighton test for the three BMI groups. Lastly, Pearson product-moment correlation coefficients were computed to examine the relationships between cardiorespiratory fitness, muscular strength, joint mobility, flexibility and BMI variables. Level of significance was established at *p* ≤ 0.05.

## 3. Results

### 3.1. Participants’ Characteristics

Table 1 displays mean ± standard deviation (SD) of participants’ characteristics. 151 female adolescents were classified as normal-weight (*n* = 78), overweight (*n* = 51) and obese (*n* = 22). The average age and BMI were (14.3 ± 0.9) years and (24.3 ± 5.5) kg/m^2^, respectively.

### 3.2. The Differences between Cardiorespiratory Fitness and Measures of Musculoskeletal Fitness among the BMI Groups

Normal-weight girls demonstrated greater cardiorespiratory fitness compared to overweight and obese girls. There was no significant difference between normal-weight and overweight girls in terms of cardiorespiratory fitness. However, normal-weight girls performed significantly better than obese girls on the 20 m shuttle run test. The same trend was evident between overweight and obese girls (Table 2).

For all the measures of MSF except elbow flexion strength, a significant difference was found among the three groups (Table 2). A post hoc analysis conducted using the Bonferroni test revealed statistically significant differences among the groups in all cases, with the exception of strength of the knee extensors, where significant differences was only seen between normal-weight and overweight girls. In general, normal-weight girls had stronger knee extensors than overweight girls. Again, normal-weight girls showed greater ankle plantarflexion and dorsiflexion strength in comparison with overweight and obese girls. Both obese and overweight girls had stronger elbow extensors compared to their normal-weight peers. With regard to grip strength, overweight and obese girls outperformed normal-weight girls, and obese girls demonstrated stronger grip strength than overweight girls. Concerning flexibility, we observed significant differences between normal-weight and overweight girls, and also between obese and normal-weight girls (Table 2). With regard to joint mobility, overweight girls demonstrated increased range of motion than their normal-weight counterparts (Table 3). The chi-square analysis of self-reported musculoskeletal complaints and pain did not yield any significant differences among the groups (Table 4).

### 3.3. The Relationship between Body Mass Index, Cardiorespiratory and Musculoskeletal Fitness among the Participants

A positive relationship was observed between BMI and mean grip strength (r = 0.410, *p* < 0.01). Further, a positive relationship was observed between BMI and Beighton total score (r = 0.215, *p* < 0.001), and mean elbow extensor strength (r = 0.323, *p* < 0.01).

Significant negative relationship was found between BMI and mean knee extensor strength (r = −0.224, *p* < 0.01), mean ankle plantarflexor strength (r = −0.362, *p* < 0.01) and mean ankle dorsiflexor strength (r = −0.312, *p* < 0.01). Finally, there was a negative correlation between BMI and cardiorespiratory fitness (r = −0.336, *p* < 0.01) (Table 5).

## 4. Discussion

The objective of this study was to investigate the relationship between body mass index, cardiorespiratory and musculoskeletal fitness in a cohort of South African female adolescents. In general, we observed that about half of the participants had higher BMI scores (were either overweight or obese). Since all the female students of grades 8, 9 and 10 of the participating school were tested; this observation is worrying and should be treated seriously to minimize the burden of obesity in this setting. This finding confirms previous reports that identified high prevalence of overweight and obesity among adolescent girls in South Africa [4,5,6,7,8,9]. Though the overweight and obesity trends in the present study were comparable to those found in previous studies, our estimates for overweight (33%) and obesity (14%) were slightly higher than those found in adolescent girls with similar characteristics [4,5,6,7,8,9]. Nonetheless, our figures are still lower than what has been documented in high-income environments [44,45]. Variations in ethnicity, culture and measurement techniques could explain these differences. The relatively high prevalence of overweight and obesity in our sample can be attributed to rapid accumulation of fat-mass, which accompanies growth and development among adolescent girls [4]. Also, the cultural interpretations ascribed to excess weight (e.g., excess weight is believed to be a sign of affluence, health and beauty) in this setting could be a plausible explanation [46,47]. 

With regard to cardiorespiratory fitness, we observed that normal-weight girls demonstrated greater performance on the shuttle run test than overweight and obese peers. Lower cardiorespiratory fitness has been shown to be associated with higher body mass index in earlier research [23,24,25,26]. Decreased lower extremity muscle strength among overweight and obese girls could contribute to low cardiorespiratory fitness due to the lack of adequate muscle power required to sustain prolonged running. Moreover, decreased cardiorespiratory fitness may limit participation in physical activity, and further cause reductions in muscular strength. The low cardiorespiratory fitness demonstrated by overweight and obese girls in the present study could be attributed to physical fatigue and poor perceived physical competence. As the 20 m shuttle run test requires individuals to carry their body mass through space, we expected this task to be somewhat difficult for both overweight and obese girls.

Concerning musculoskeletal fitness, overweight and obese girls showed reduced lower extremity muscle strength and stronger upper limbs as compared to normal-weight peers. Similar results have previously been reported. It is thought that the lack of regular voluntary activation of the lower extremity muscles among children with higher BMI may explain the decreased lower extremity muscle strength [45]. It should be noted that one important factor that might lead to poor performance on running-based aerobic capacity test is reduced lower extremity strength. This underscores the need to develop an integrated approach towards addressing the multifaceted problems of obesity in this vulnerable group. It may be prudent to promote physical activity by improving lower extremity muscular strength and dynamic control. This could potentially be useful for weight control and might increase lean body mass [48,49]. 

Furthermore, we observed that the elbow extensors of overweight and obese girls were stronger than those of that of normal-weight girls. This finding is in agreement with previous data [29]. It is possible that girls with increased BMI might find alternative or compensatory mechanisms to move their bodies without having exert the lower extremity musculature. For example, they may want to use their elbow extensors to push themselves out of a chair or hold onto a rail to propel themselves up a flight of stairs instead of accomplishing these tasks with their lower extremity muscles. Thus, with these alternative movement adaptations, they could achieve everyday movement goals without engaging the weaker lower extremity muscles. Frequent use of upper extremity muscles in goal-directed tasks might be a plausible explanation for the increased upper extremity strength. Also, we found that overweight and obese girls exhibited increased range of motion in their joints than normal-weight girls. Related to the frequency of musculoskeletal complaints, we observed no differences in the frequency and/or severity of joint or muscle pain among the groups. Our findings indicate that overweight and obese girls have about 33% and 15% reduction in mean ankle and knee forces respectively, compared to normal-weight girls. This suggests that overweight and obese girls, who would naturally require more power to transport their body mass from one place to another, might have to do so with less force. Thus, their excess load has to be supported and transported by relatively weaker muscles. Ankle dorsiflexors and knee extensors are the most important force generating muscles required for daily tasks such as walking, stair-climbing and running. Since overweight and obese girls demonstrated decreased strength in these muscles, participating in weight bearing tasks might be problematic. Though excess body mass may stimulate bone growth, greater loads might damage musculoskeletal tissues over time, and increase risks for degenerative joint diseases such as osteoarthritis [50]. Our findings on the low levels of lower extremity muscle strength confirms that reported in previous studies [51,52]. This finding emphasises the need for regular assessments of functional muscle strength among overweight or obese adolescents. In addition, exercise strategies involving activities that would ensure gradual functional loading of these joints may be a useful approach for promoting physical activity in this population.

Though we were unable to confirm the harmful effects of overloading on musculoskeletal structures, we presumed that reduced lower extremity muscle strength and hypermobility may be detrimental to musculoskeletal health among overweight and obese girls. Hypermobile joints, increased flexibility and reduced strength might be correlates of localized motor control dysfunction (around the hip, knee and ankle joints), and could synergistically limit one’s capacity to engage in tasks with high dynamic balance control demands. Additionally, it is likely that these factors might interact together to cause recurring sprains, joint damage and fractures [53,54]. Without effective intervention, overweight and obese girls might continually experience a vicious cycle of reduced lower extremity muscle strength, low cardiorespiratory fitness, and physical inactivity.

The cross-sectional nature of the present study does not allow causal inferences to be made about the relationship between the measured variables. More longitudinal data and randomised controlled studies are urgently needed to determine causality, as many available studies are largely observational. Another limitation is that due to logistical constraints, we were unable to measure maturation indexes. It might be useful to investigate the association between these variables and measures of sexual maturation in girls. Again, as the study involved adolescent girls only, the findings cannot be generalised to boys. Participants of the present study were black South African girls, and so extrapolation of the results to other ethnic populations with dissimilar characteristics should be done with utmost caution. Further, the use of BMI to define overweight and obesity has inherent limitations. Although BMI is a widely used index in research involving children and adolescents, it is not considered as a direct indicator of body composition. Future research should employ more accurate field-based measures such as skinfold or waist circumference assessments. 

## 5. Conclusions

Overweight and obese girls have decreased cardiorespiratory and musculoskeletal fitness as compared to normal-weight peers. More research efforts should be directed towards developing interventions with the goal of improving these important components of physical fitness in this demographic group. This might prevent or delay the progression of long-term health complications. Given that overweight and obese female adolescents demonstrate decreased lower extremity strength and cardiorespiratory fitness, we propose that light-intensity, meaningful and fun exercises involving the use of everyday tasks with optimal joint loading should be explored. Due to the functional problems identified in this group, vigorous intensity activities (e.g., running) and complex sports might be too challenging and could decrease enthusiasm. Hence, using familiar activities like walking over obstacles, controlled lunges, and motor skills instruction, might be deemed enjoyable and could increase compliance. Thus, an exercise approach that allows for adequate development of motor competence, strength and cardiorespiratory fitness in a motivating environment may prove to be useful for preparing these girls for usual physical education programmes.

## Figures and Tables

**Table 1:** Demographic and anthropometric characteristics of participants.

Groups					
Variables	Normal-Weight (*n* = 78)	Overweight (*n* = 51)	Obese (*n* = 22)	ANOVA (*F*)	*p*-Value
Age (years)	14.3 ± 0.9	14.4 ± 0.9	14.2 ± 0.9	0.455	0.636
Height (cm)	158.2 ± 6.8	157.9 ± 6.3	157.8 ± 6.1	0.050	0.951
Weight (kg)	51.1 ± 6.6	64.9 ± 5.7 *	85.8 ± 14.1 +,#	177.203	0.001
BMI (kg/m^2^)	20.4 ± 2.0	26.0 ± 1.5 *	34.4 ± 5.1 +,#	277.057	0.001

Data are presented as (Means ± SD); BMI-Body Mass Index. * *p* < 0.05, Normal vs overweight, + *p* < 0.05, Obese vs. normal, # *p* < 0.05, Overweight vs. obese (One-way ANOVA; Bonferroni post hoc test).

**Table 2:** Comparison of cardiorespiratory and musculoskeletal fitness among groups.

Groups					
Variables	Normal-Weight (*n* = 78)	Overweight (*n* = 51)	Obese (*n* = 22)	ANOVA (*F*)	*p*-Value
**Cardiorespiratory fitness**					
20 m shuttle run test (level)	2.6 ± 1.3	2.2 ± 1.1	1.4 ± 0.6 +,#	9.878	0.001
**Muscular strength**					
Knee extension (Right leg) (N)	187.2 ± 68.9	159.3 ± 24.9 *	159.3 ± 27.9	5.275	0.006
Knee extension (Left leg) (N)	176.6 ± 63.4	154.5 ± 26.2 *	158.2 ± 29.5	3.437	0.035
Ankle plantarflexion (Right leg) (N)	160.5 ± 79.7	108.4 ± 28.0 *	108.7 ± 22.5 #	14.019	0.001
Ankle plantarflexion (Left leg) (N)	162.0 ± 81.5	109.1 ± 22.4 *	113.8 ± 23.2 #	13.549	0.001
Ankle dorsiflexion (Right leg) (N)	167.5 ± 70.9	127.5 ± 20.9 *	127.4 ± 20.5 #	10.677	0.001
Ankle dorsiflexion (Left leg) (N)	162.3 ± 69.6	125.5 ± 21.4 *	124.9 ± 23.1 #	9.365	0.001
Elbow flexion (Right arm) (N)	138.4 ± 27.2	128.8 ± 16.0	128.6 ± 20.5	3.307	0.039
Elbow flexion (Left arm) (N)	138.4 ± 26.7	138.9 ± 21.1	138.6 ± 21.3	0.007	0.993
Elbow extension (Right arm) (N)	91.1 ± 17.5	97.8 ± 23.9	109.4 ± 29.8 #	6.273	0.002
Elbow extension (Left arm) (N)	90.0 ± 19.4	99.4 ± 16.7 *	111.7 ± 30.7 #	10.341	0.001
Grip strength (Right arm) (N)	20.7 ± 3.7	24.5 ± 4.3 *	24.8 ± 5.0 #	17.317	0.001
Grip strength (Left arm) (N)	20.7 ± 4.1	23.8 ± 4.6 *	25.0 ± 4.9 #	12.143	0.001
**Flexibility**					
Sit-and-reach test (cm)	6.3 ± 5.9	10.5 ± 6.2 *	12.1 ± 6.5 +	11.849	0.001

Data are presented as (Means ± SD); N-Newton. * *p* < 0.05, Normal vs overweight, + *p* < 0.05, Obese vs. normal, # *p* < 0.05, Overweight vs. obese (One-way ANOVA; Bonferroni post hoc test).

**Table 3:** Comparison of joint mobility among normal-weight, overweight and obese girls.

Groups			
Variables	Normal-Weight (*n* = 78)	Overweight (*n* = 51)	Obese (*n* = 22)
Dorsiflexion of the fifth metacarpophalangeal joint (Right arm) (degrees)	77.7 ± 15.9	81.8 ± 16.9	77.5 ± 17.2
Dorsiflexion of the fifth metacarpophalangeal joint (Left arm) (degrees)	80.9 ± 15.8	86.6 ± 15.2	83.6 ± 15.7
Hyperextension of the elbow (Right arm) (degrees)	8.1 ± 6.8	11.2 ± 6.3 *	8.8 ± 5.7
Hyperextension of the elbow (Left arm) (degrees)	8.3 ± 6.8	11.7 ± 6.6 *	9.8 ± 7.8
Hyperextension of the knee (Right leg) (degrees)	7.5 ± 7.4	6.0 ± 4.9	9.2 ± 6.6
Hyperextension of the knee (Left leg) (degrees)	6.9 ± 5.3	8.1 ± 6.1	11.4 ± 12.9 #
Apposition of the thumb to the flexor side of the forearm (Right arm) Yes No	28 (35.9) 50 (64.1)	27 (40.3) 24 (47.1)	12 (54.5) 10 (45.5)
Apposition of the thumb to the flexor side of the forearm (Left arm) Yes No	24 (30.8) 54 (69.2)	28 (54.9) 22 (43.1)	13 (59.1) 9 (40.9)
Forward flexion of the trunk with the knee straight and hands on the floor Yes No	14 (17.9) 64 (82.1)	13 (25.5) 38 (74.5)	9 (40.9) 13 (59.1)
Beighton total score	3.0 ± 2.2	4.2 ± 2.2 *	4.3 ± 2.5

Data are presented as (Means ± SD) or n (%). * *p* < 0.05, Normal vs overweight, # *p* < 0.05, Overweight vs. obese (one-way ANOVA; Bonferroni post hoc test).

**Table 4:** Comparison of musculoskeletal complaints among normal-weight, overweight and obese girls.

Groups					
Variables	Normal Weight (*n* = 78)	Overweight (*n* = 51)	Obese (*n* = 22)	*χ*^2^ (df = 2)	*p*-Value
Joint pain Yes No	12 (15.4) 66 (84.6)	5 (9.8) 46 (90.2)	5 (18.2) 18 (81.8)	1.195	0.550
Muscle pain Yes No	17 (21.8) 61 (78.2)	7 (13.7) 44 (86.3)	7 (31.8) 15 (68.2)	3.242	0.198
Pain severity 0 2 4 6 8 10	55 (70.5) 12 (15.4) 8 (10.3) 1 (1.3) 2 (2.6) 0	41 (80.4) 2 (3.9) 6 (11.8) 2 (3.9) 0 0	14 (63.6) 5 (22.7) 2 (9.1) 1 (4.5) 0 0	9.193	0.514

Data are presented as *n* (%). Note. *n*-number of participants, %-percentage.

**Table 5:** Pearson Correlation matrix for body mass index, cardiorespiratory fitness, and measures of musculoskeletal fitness.

Correlations	1	2	3	4	5	6	7	8	9	10
BMI (kg/m^2^)	-									
Beighton total score	0.215 **	-								
Sit-and-reach score (cm)	0.310 **	0.241 **	-							
Mean elbow flexors strength (N)	−0.090	−0.119	−0.057	-						
Mean elbow extensors strength (N)	0.323 **	0.055	0.124	0.374 **	-					
Mean knee extensors strength (N)	−0.224 **	−0.278 **	−0.248 **	0.723 **	0.251 **	-				
Mean ankle plantarflexors strength (N)	−0.362 **	−0.365 **	−0.274 **	0.613 *	0.219 **	0.825 **	-			
Mean ankle dorsiflexors strength (N)	−0.312 **	−0.303 **	−0.258 **	0.697 **	0.232 **	0.856 **	0.872 **	-		
mean Grip strength (N)	0.410 **	0.117	0.236 **	0.030	0.225 **	−0.130	−0.217 **	−0.210 **	-	
20 m shuttle run test (level)	−0.336 **	0.062	0.004	0.102	−0.107	0.036	0.059	0.113	−0.051	-

Abbreviations: BMI-Body Mass Index, N-Newton. ** Correlation is significant at 0.01 level (2-tailed), * Correlations are significant at 0.05 level (2-tailed).
